# Hydroxytyrosol Acetate Inhibits Vascular Endothelial Cell Pyroptosis via the HDAC11 Signaling Pathway in Atherosclerosis

**DOI:** 10.3389/fphar.2021.656272

**Published:** 2021-04-23

**Authors:** Feng Yao, Zhen Jin, Xiaohan Lv, Zihan Zheng, Hongqian Gao, Ying Deng, Yizhen Liu, Lifang Chen, Weirong Wang, Jianyu He, Jianli Gu, Rong Lin

**Affiliations:** ^1^ School of Pharmacy, Xi’an Jiaotong University Health Science Center, Xi’an, China; ^2^ Department of Pharmacology, Xi’an Jiaotong University Health Science Center, Xi’an, China; ^3^ Department of Medical Laboratory Animal Science, Xi’an Jiaotong University Health Science Center, Xi’an, China; ^4^ Department of Pathology, Xi’an Jiaotong University Health Science Center, Xi’an, China; ^5^ Xi'an NO.3 Hospital, Xi'an, China

**Keywords:** hydroxytyrosol acetate, atherosclerosis, HDAC11, pyroptosis, vascular endothelial cells

## Abstract

Hydroxytyrosol acetate (HT-AC), a natural polyphenolic compound in olive oil, exerts an anti-inflammatory effect in cardiovascular diseases (CVDs). Pyroptosis is a newly discovered form of programmed inflammatory cell death and is suggested to be involved in the atherosclerosis (AS) process. However, the effect of HT-AC on vascular endothelial cell pyroptosis remains unknown. Thus, we aimed to investigate the effect of HT-AC on vascular endothelial cell pyroptosis in AS and related signaling pathways. *In vivo* studies showed that HT-AC alleviated the formation of atherosclerotic lesions and inhibited pyroptosis in the aortic intima of ApoE^−/−^ mice fed a high-fat diet (HFD) for 12 weeks. *In vitro*, we found that HT-AC treatment of human umbilical vein endothelial cells (HUVECs) alleviated tumor necrosis factor-alpha (TNF-α)-induced pyroptosis by decreasing the number of PI positive cells, decreasing the enhanced protein expressions of activated caspase-1 and gasdermin D (GSDMD), as well as by decreasing the release of pro-inflammatory interleukin (IL)-1β and IL-6. Besides, HT-AC down-regulated HDAC11 expression in the aortic intima of HFD-fed ApoE^−/−^ mice and TNF-α-stimulated HUVECs. To determine the underlying mechanism of action, molecular docking and drug affinity responsive target stability (DARTS) were utilized to identify whether HDAC11 protein is a target of HT-AC. The molecular docking result showed good compatibility between HT-AC and HDAC11. DARTS study's result showed that HDAC11 protein may be a target of HT-AC. Further study demonstrated that knockdown of HDAC11 augmented the inhibition of HT-AC on pyroptosis in TNF-α-stimulated HUVECs. These findings indicate that HT-AC might prevent vascular endothelial pyroptosis through down-regulation of HDAC11 related signaling pathway in AS.

## Introduction

Atherosclerosis (AS) is a chronic inflammatory disease that is initiated by endothelial dysfunction and structural alterations and involves chronic inflammation of the vascular wall (Weber and Noels, 2011). Previous studies have shown that endothelial cell death is a crucial and initial stage in the process of AS (Wu et al., 2018; Xing et al., 2020). Pyroptosis is a newly discovered form of programmed inflammatory cell death differing from apoptosis and necrosis and suggested to be involved in AS process, which features gasdermin family mediated pore formation on the plasma membrane as well as the release of pro-inflammatory intracellular contents including interleukin (IL)-1β, IL-18 and High Mobility Group Box 1 (HMGB1) (Jia et al., 2019; Wang et al., 2020). Thus, the inhibition of endothelial cell death especially pyroptosis is of great significance to prevent AS. Hydroxytyrosol acetate (HT-AC) is the polyphenolic compound, which is present in the olive oil of the fruit and leaf of the olive (Lisete-Torres et al., 2012). Olive oil phenolic compounds can be used to prevent and treat cardiovascular diseases (CVDs) (Bulotta et al., 2014). Accumulating evidence indicates that HT-AC has antioxidant (Gordon et al., 2001), antiplatelet aggregating (González-Correa et al., 2008), and anti-inflammatory activities (Rosillo et al., 2015). In our previous study, we demonstrated that HT-AC had an anti-inflammatory effect in hypercholesterolemic mice and tumor necrosis factor-alpha (TNF-α)-stimulated human umbilical vein endothelial cells (HUVECs) (Yao et al., 2019). However, the underlying mechanism of HT-AC on inflammation is still under investigation. Since pyroptosis is involved in the inflammatory process of AS (Chang et al., 2013), we hypothesize that HT-AC might play a role in inflammation by regulating pyroptosis in endothelial cells. Thereby, we conducted this study to prove it.

Histone deacetylase 11 (HDAC11) is a class IV histone deacetylase, which is one of the most recently identified members of the HDAC family (Nutsford et al., 2019). HDAC11 has been shown to play a role in a variety of diseases including kidney ischemia and reperfusion (I/R) injury (Kim et al., 2013), cancer (Yue et al., 2020) and CVDs (Fan et al., 2018; Yuan et al., 2018). A study showed that HDAC11 knockout (HDAC11^−/−^) attenuated apoptosis, oxidative stress and inflammation in the heart of fructose-fed mice (Fan et al., 2018). Another study demonstrated that HDAC11 compromised the vascular endothelial barrier function by suppressing the expression of Ve-cadherin (Zhang and Ge, 2017). Besides, it has been reported that treatment of the HDAC11 inhibitor attenuated vascular injury in mice (Zhou et al., 2017). These studies suggested that HDAC11 was involved in CVDs. However, whether the regulation of HT-AC on the vascular endothelial cell pyroptosis is mediated by HDAC11 remains unknown.

Therefore, in the present study, we first determined the effects of HT-AC on the formation of atherosclerotic lesions and pyroptosis in the aortic intima of high-fat diet (HFD)-fed ApoE^−/−^ mice for 12 weeks. Then, we examined the effect of HT-AC on pyroptosis in TNF-α-stimulated HUVECs and whether this effect of HT-AC was mediated by HDAC11 to elucidate its antiatherogenic mechanism.

## Materials and Methods

### Materials

HT-AC (purity>98%) was purchased from APP-Chem Bio (Xi’an, China). Oil Red O staining kit and propidium iodide (PI) solution were purchased from Solarbio (Beijing, China). Mouse TNF-α and IL-1β enzyme-linked immunosorbent assay (ELISA) kit and human IL-6 ELISA kit were products of TBhealthcare (Foshan, China). TNF-α was obtained from PeproTech (Rocky Hill, New Jersey, United States of America). A CCK8 assay kit was obtained from biosharp (Hefei, China). Human IL-1β ELISA, lactate dehydrogenase (LDH) cytotoxicity assay kit, caspase-1 activity assay kit, 4′, 6-diamidino-2-phenylindole (DAPI), and Hoechchst 33342 staining solution for live cells were purchased from Beyotime (Haimen, China). Pro-caspase-1 and cleaved caspase-1 (caspase-1 p20) antibodies were obtained from Wanleibio (Shenyang, China). Antibodies against HDAC11 and CD31 were ordered from Abcam (Cambridge, Massachusetts, United States of America). Gasdermin D (GSDMD) antibody, cleaved GSDMD (Asp275) (GSDMD-N) rabbit mAb, mouse anti-β-actin, goat anti-mouse and anti-rabbit IgG-HRP antibodies, and goat anti-rabbit IgG (H + L) were purchased from Cell Signaling Technology Inc. (Beverly, Massachusetts, United States of America). Protease inhibitor cocktail and pronase were products of Roche (Mannheim, Germany). The Bradford protein assay kit was obtained from Pierce (Rockford, Illinois, United States of America). Lipofectamine 2000 transfection reagent and TRIzol reagent were purchased from Invitrogen (Carlsbad, California, United States of America). All-in-One cDNA Synthesis SuperMix and 2 × SYBR qPCR Master Mix were purchased from Bimake (Houston, Texas, United States of America). Fetal bovine serum (FBS) was obtained from Gibco (Grand Island, New York, United States of America). All these reagents were of analytical grade unless otherwise specified.

### Animals and Establishment of Atherosclerosis Model

ApoE^−/−^ mice in the C57/BL6 background were purchased from The Jackson Laboratory (Bar Harbor, ME). Eight-week-old male ApoE^−/−^ mice were housed in a SPF facility (12 h light/dark cycle). The mice were randomly divided into four groups (ten animals each): the normal diet (ND) group, the normal diet group treated with HT-AC (ND + HT-AC), the HFD group, and the HFD group treated with HT-AC (HFD + HT-AC). The animals of the HFD group were maintained on diet with a Western diet consisting of 21% fat and 0.15% cholesterol (Huafukang, Beijing, China) for 12 weeks to induce atherosclerosis. HT-AC was dissolved in sterile saline, then mice were gavaged with HT-AC at a dosage of 20 mg/kg/day for 12 weeks. After 12 weeks of treatment, all mice were fasted overnight (12 h), and then anesthetized with intraperitoneal 50 mg/kg of sodium pentobarbital and sacrificed.

### Immunohistology of Atherosclerotic Lesions

The aortic root along with the basal portion of the heart was fixed with 4% paraformaldehyde, followed by embedding in optimum cutting temperature (OCT) compound (SAKURA, United States of America), and was then cut cross-sectionally into 7 μm-thick sections. Hematoxylin and eosin (H&E) staining and Oil red O staining were performed to show atherosclerotic lesions of the aortic root and the lipid deposition of atherosclerotic lesions. Besides, immunostaining was performed on the aortic root for the expressions of cleaved caspase-1 and HDAC11. CD31 was used as an endothelial marker. Images were captured using a fluorescence microscope (Olympus TL4, Japan).

### Cell Culture and Treatment

A human umbilical vein endothelial cell line (CRL-1730 cells) was obtained from the American Type Culture Collection (ATCC, Manassas, United States of America). In brief, HUVECs were grown in DMEM/F-12 medium supplemented with 10% FBS and 100 U/ml penicillin-streptomycin in a humidified atmosphere of 5% CO_2_ at 37°C. When cells were grown to confluence, the culture medium was replaced with a serum-free medium for 24 h of incubation prior to experimental use. To determine whether HT-AC inhibited endothelial cell pyroptosis, cells were pretreated with different concentrations of HT-AC (25, 50 and 100 μmol/L) for 1 h and then incubated with TNF-α (40 ng/ml) for 12 h. To further detect whether the effect of HT-AC on pyroptosis was mediated by HDAC11, HDAC11 small interfering RNA (siRNA) was used. HDAC11 siRNA and negative control (NC) siRNA were chemically synthesized by Shanghai GenePharma Corporation (SGC, China). HUVECs were transfected with HDAC11 siRNA or NC siRNA using Lipofectamine 2000 according to the manufacturer's instructions. After 48 h, cells were pretreated with HT-AC (50 μmol/L) for 1 h and then incubated with TNF-α (40 ng/ml) for 12 h. HT-AC was dissolved in sterile saline.

### Western Blotting

Total protein was extracted from the snap-frozen thoracic aorta of mice (20–30 mg) and HUVECs with RIPA lysis buffer containing protease inhibitor cocktail. The protein concentrations were determined by using the Bradford protein assay kit. Equal amounts of protein lysates were separated by SDS-PAGE gels and transferred to polyvinylidene difluoride membranes followed by a block with 5% skimmed milk at room temperature for 2–3 h. Subsequently, the membranes were incubated with primary antibodies against pro-caspase-1 (diluted 1:1,000), cleaved caspase-1 (diluted 1:1,000), GSDMD (diluted 1:1,000), GSDMD-N (diluted 1:1,000), HDAC11 (diluted 1:200) or *β*-actin (diluted 1:5,000) at 4°C overnight. After washing three times with tris-buffered saline and Tween 20 (TBST), the membranes were incubated with goat anti-rabbit IgG-HRP antibody (diluted 1:5,000) or goat anti-mouse IgG-HRP antibody (diluted 1:5,000) for 1–2 h. Then the membranes were washed three times with TBST and the enhanced chemiluminescent substrate was used for detection. The intensity of protein bands was analyzed by Lane 1D software (Sage Creation Science Co., China).

### Cytokine Analysis by ELISA

Blood samples were obtained by penetrating the retro-orbital sinus in mice, and the serum was separated by centrifugation at 3,000 rpm for 15 min at 4°C. All samples were stored at −80°C until analysis. According to the manufacturer's instructions, concentrations of TNF-α and IL-1β in the serum of ApoE^−/−^ mice and concentrations of IL-1β and IL-6 in cellular supernatant of HUVECs were detected by ELISA assay.

### Cell Viability Assay

The cell viability was determined by a CCK8 assay. HUVECs were seeded in 96-well plates in complete medium and treated with different concentrations of HT-AC (12.5, 25, 50, 100 and 200 μmol/L) for 13 h, subsequently treated with 10 μl CCK8 reagent for 4 h. And then absorbance measurements were taken at 450 nm using a microplate reader (Thermo Fisher, United States of America).

### Caspase-1 Activity Analysis

HUVECs were seeded onto a 6-well microplate with three replicate wells for each condition and cultured overnight to allow cells to attach. After the indicated treatments, cells were collected and caspase-1 activity was assayed using the caspase-1 activity assay kit according to the manufacturer’s instructions.

### Measurement of LDH Release

HUVECs were plated in 48-well plates. Cell supernatants were collected and cellular debris was removed by centrifugation at 1,500 rpm for 5 min. Supernatant LDH levels were measured using the cytotoxicity detection LDH kit according to the manufacturer’s instruction.

### Hoechst 33342/PI Fluorescent Staining

Double-fluorescent staining with Hoechst 33342 and PI was used to assess the formation of membrane pores during pyroptosis. The cells were stained with Hoechst 33342 staining solution for live cells and 2 μg/ml PI for 20 min in a humidified atmosphere of 5% CO_2_ at 37°C. Then the cells were washed three times with PBS. The fluorescence of Hoechst 33,342 was detected by a fluorescence microscope (Olympus TL4, Japan) at the excitation wavelength of 346 nm and the emission wavelength of 460 nm, and the fluorescence of PI was detected at the excitation wavelength of 535 nm and the emission wavelength of 615 nm.

### Immunofluorescence

The HUVECs were fixed with 4% paraformaldehyde for 30 min, subsequently permeabilized with 0.3% Triton X-100 for an additional 30 min, and then blocked with normal goat serum for 1 h at room temperature. To determine the effect of HT-AC on HDAC11 expression and localization, the samples were incubated with anti-HDAC11 antibody (diluted 1:100) overnight at 4°C, followed by incubation with a goat anti-rabbit IgG (H + L) antibody (diluted 1:200) for 2 h at room temperature. Nuclei were stained with DAPI (1 μg/ml) dye and observed by a fluorescence microscope (Olympus TL4, Japan).

### RNA Extraction and Real-Time PCR

RNA was extracted from the thoracic aorta of mice and cultured HUVECs using TRIzol reagent. RNA quality and integrity were detected using a NanoDrop ND1000 and determined via the A260/A280 ratio. And then the RNA was reversely transcripted to cDNA using All-in-One cDNA Synthesis SuperMix following the manufacturer's instructions. Quantitative real-time PCR was performed with 2 × SYBR Green qPCR Master Mix by StepOnePlus™ Real-Time PCR System (Thermo Fisher, Massachusetts, United States of America). Relative changes in mRNA levels were analyzed by the ΔΔCT method using GAPDH as a control. The sequences of primer pairs are as follows: Mouse HDAC11: forward 5′-GCC​CTG​GAT​CTG​CTC​CAA​CTA​C-3′,reverse 5′-AAC​TGG​TGA​CTC​TGG​CAT​CCT​C-3’;Mouse GAPDH: forward 5′-TGT​GTC​CGT​CGT​GGA​TCT​GA-3′,reverse 5′-TTG​CTG​TTG​AAG​TCG​CAG​GAG-3’;Human HDAC11: forward 5′-GAT​GTC​TAC​AAC​CGC​CAC​ATC​TAC​C-3′,reverse 5′- CCT​GCA​TTG​TAT​ACC​ACC​ACG​TC-3’;Human GAPDH: forward 5′-GGA​GCG​AGA​TCC​CTC​CAA​AAT-3′,reverse 5′-GGC​TGT​TGT​CAT​ACT​TCT​CAT​GG-3’.

### HDAC11 Protein Homology Modeling and Molecular Docking

The amino acid sequence of HDAC11 was retrieved from the protein database of NCBI (NP_079103.2). The crystal structure of human HDAC11 was built and validated using a fully automated protein structure homology-modeling tool at the Swiss-Model server (http://swissmodel.expasy.org). It has been shown that a good quality model would be expected to have over 90% in the most favored regions (Laskowski et al., 1996). The Surflex-Dock module of SYBYL X-2.0 (Tripos Inc., Saint Louis, MO, United States of America) was performed for the docking calculations with default parameters.

### Drug Affinity Responsive Target Stability (DARTS) Analysis

Here, DARTS methods were used to identify whether HDAC11 protein is a target of HT-AC. Total protein was extracted from HUVECs with M-PER reagent (Thermo Scientific) containing a 1× protease inhibitor cocktail. The protein concentration of cell lysate was determined by using the Bradford protein assay kit. Then cell lysate was divided into identical aliquots of 50 μl, which was incubated with different concentrations of HT-AC (10, 100 and 1,000 μmol/L) or sterile saline for 2 h at 4°C. After incubation with the HT-AC is complete, each sample added 2 μl pronase solutions at different mass ratios of pronase to protein (1:100, 1:500, 1:1,000, 1:2,500 and 1:5,000). Then each sample was incubated with pronase for 20 min at room temperature, and each digestion reaction was stopped by adding 2 μl of 20 × protease inhibitor cocktail on ice for 10 min. Then the samples were subjected to SDS–PAGE for coomassie staining and Western blotting. Anti-HDAC11 (diluted 1:200) and anti-β-actin (diluted 1:5,000) antibodies were used as the primary antibodies.

### Statistical Analysis

All quantitative data are presented as the mean ± SEM from three independent experiments. Statistical analysis was undertaken using GraphPad Prism (GraphPad Software, La Jolla, CA, United States of America). Multiple comparisons were assessed by one-way analysis of variance. Statistical significance was ascribed to a *p* value <0.05.

## Results

### HT-AC Attenuated the Formation of Atherosclerotic Lesions in ApoE^−/−^ Mice

**FIGURE 1:**
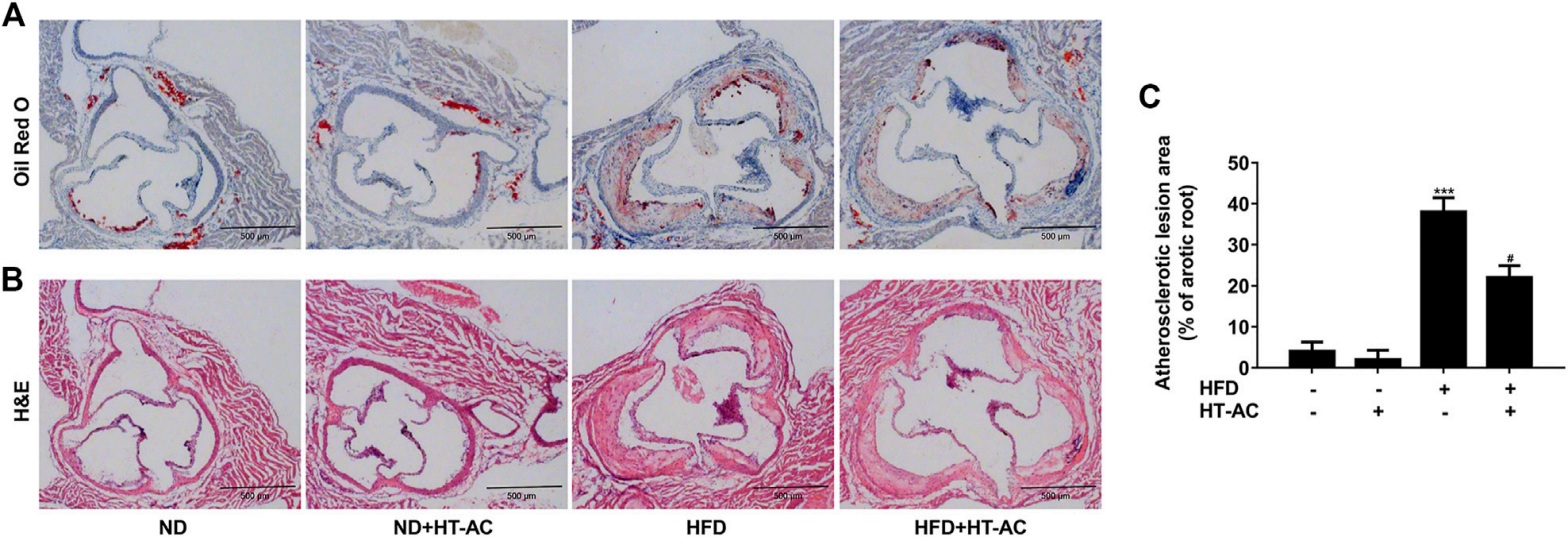
HT-AC attenuated the formation of atherosclerotic lesions in ApoE^−/−^ mice. ApoE^−/−^ mice with ND or HFD with or without HT-AC treatment (20 mg/kg/day) for 12 weeks **(A)** Representative images showing the lipid deposition of aortic root sections with Oil red O staining, ×100 **(B)** Representative images showing the atherosclerotic lesions of aortic root sections with H&E staining, ×100. Scale bar indicates 500 μm **(C)** The atherosclerotic lesion area of aortic root sections was analyzed. ***p < 0.001 vs. ND group; ^#^ *p* < 0.05 vs. HFD group. Results are expressed as mean ± SEM (*n* = 10).

To examine the possible impact of HT-AC on AS, we performed H&E and Oil Red O staining in histological sections of the aortic sinus of the ApoE^−/−^ mice. Our results showed that severe atherosclerosis was successfully induced in our model, while 12 weeks of HT-AC treatment decreased the lipid deposition(Figure 1A) and plaque formation in ApoE^−/−^ mice fed with HFD (Figures 1B,C). By comparison, no significant changes were noticed in the mice treated with ND. These results suggested that HT-AC attenuated the formation of atherosclerotic lesions in HFD-fed ApoE^−/−^ mice.

### HT-AC Inhibited Pyroptosis in the Aortic Intima of HFD-Fed ApoE^−/−^ Mice

**FIGURE 2:**
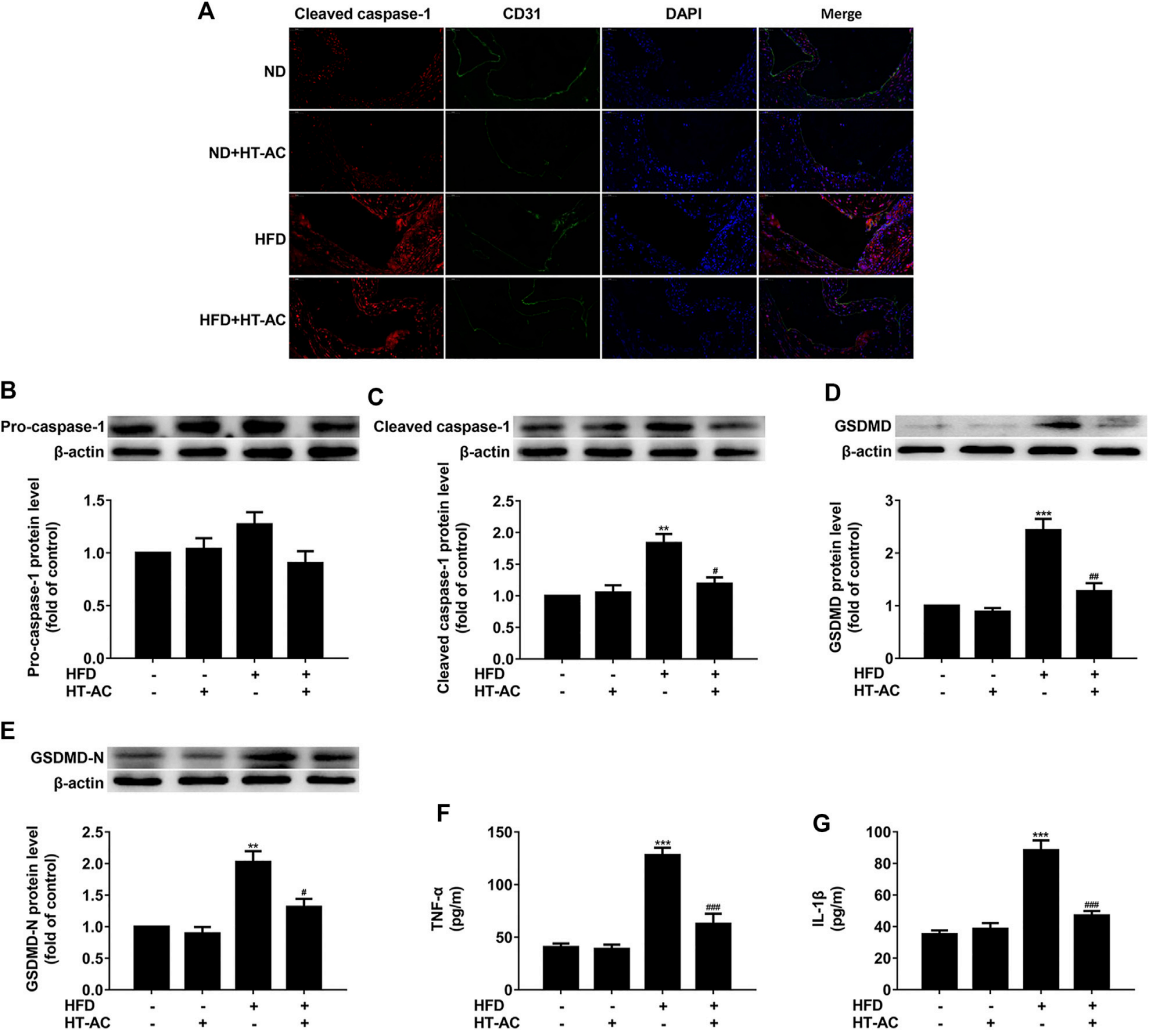
HT-AC inhibited pyroptosis in the aortic intima of HFD-fed ApoE^−/−^ mice **(A)** Frozen sections of aortic root were stained for activated caspase-1 (cleaved caspase-1) (stained in red). The nuclei were stained blue with DAPI. CD31 (stained in green) was used as an endothelial marker. Scale bar indicates 50 μm **(B–E)** The protein expressions of pro-caspase-1, cleaved caspase-1, GSDMD and GSDMD-N were determined by Western blotting **(F,G)** The concentrations of TNF-α and IL-1β in the serum were determined by ELISA. **p < 0.01, ***p < 0.001 vs. ND group; ^#^ *p* < 0.05, ^##^ *p* < 0.01, ^###^ *p* < 0.001 vs. HFD group. Results are expressed as mean ± SEM (*n* = 10).

Compared with the ND group, immunofluorescent double staining of the aortic sinus of cleaved caspase-1 and CD31 (an endothelial cell marker) revealed that caspase-1 activation (cleaved caspase-1 intensity) was significantly increased in the HFD group, while HT-AC significantly suppressed such an increase (Figure 2A). Western blotting results further showed that the protein expressions of cleaved caspase-1, GSDMD and GSDMD-N in the thoracic aorta of HFD-fed ApoE^−/−^ mice were significantly increased as compared with the ND group, while HT-AC significantly suppressed the enhanced protein expressions of cleaved caspase-1, GSDMD and GSDMD-N (Figures 2B-E). In addition, concentrations of TNF-α and IL-1β in the serum of HFD-fed ApoE^−/−^ mice were significantly increased as compared with the ND group, while HT-AC significantly suppressed concentrations of TNF-α and IL-1β in the serum of HFD-fed ApoE^−/−^mice (Figures 2F,G). Our findings suggested that HT-AC suppressed pyroptosis in the aortic intima of HFD-fed ApoE^−/−^ mice.

### HT-AC Inhibited TNF-α-Induced Pyroptosis in HUVECs

**FIGURE 3:**
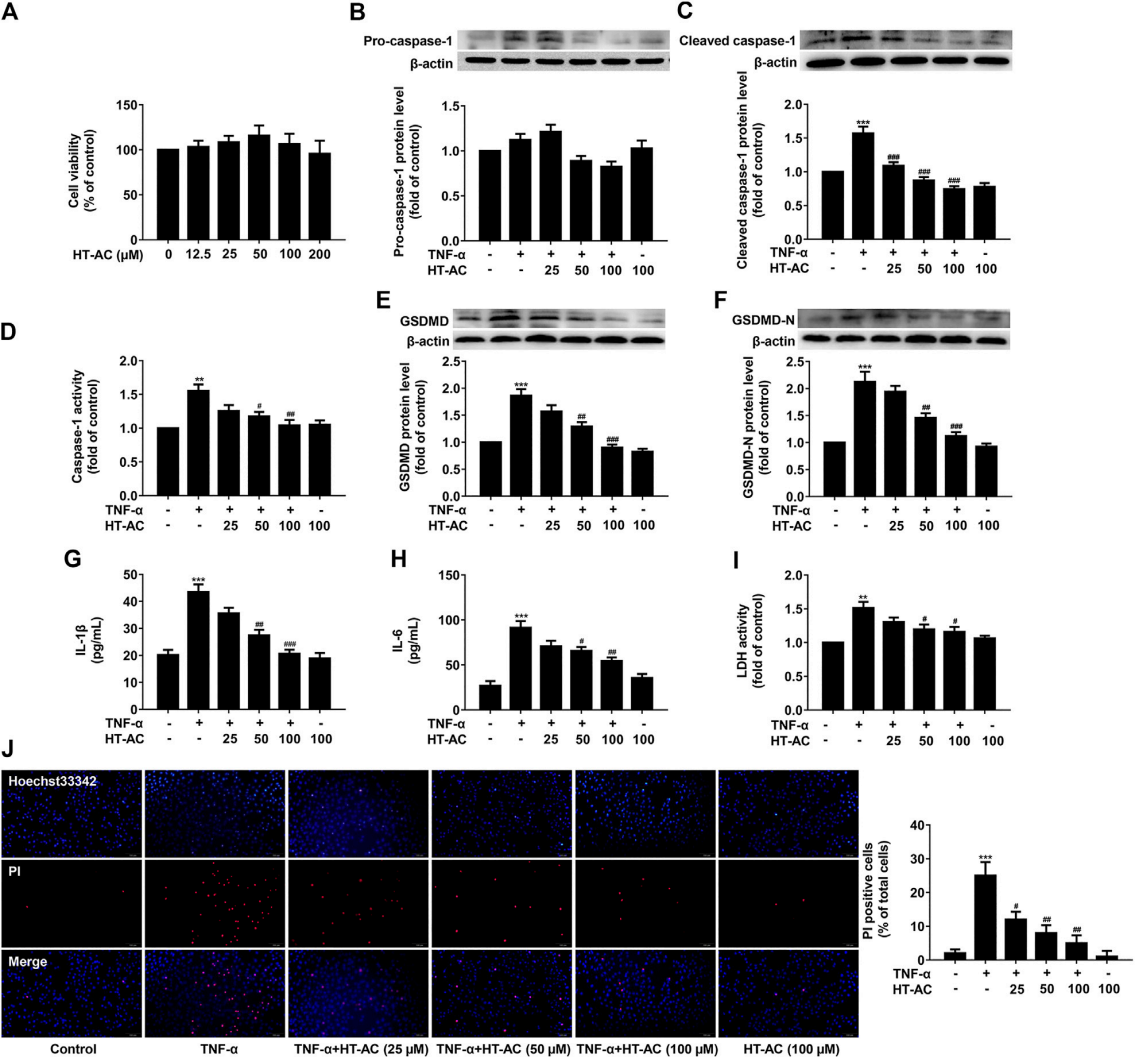
HT-AC inhibited pyroptosis in TNF-stimulated HUVECs **(A)** The cell viability was measured after incubation with various concentrations of HT-AC (12.5, 25, 50, 100 and 200 μmol/L) for 13 h using the CCK8 assay. The HUVECs were treated with different concentrations of HT-AC (25, 50 and 100 μmol/L) for 1 h, and then stimulated with TNF-α (40 ng/ml) for 12 h **(B,C)** The protein expressions of pro-caspase-1 and cleaved caspase-1 were determined by Western blotting **(D)** Caspase-1 activity was assayed using the caspase-1 activity assay kit **(E,F)** The protein expressions of GSDMD and GSDMD-N were determined by Western blotting **(G,H)** The concentrations of IL-1β and IL-6 in cellular supernatant of HUVECs were detected by ELISA assay **(I)** The LDH release was evaluated with a cytotoxicity detection LDH kit **(J)** Double-fluorescent staining with PI (red) and Hoechst 33,342 (blue) was used to assess the formation of membrane pores during pyroptosis, ×200. Scale bar indicates 100 μm **p < 0.01, ***p < 0.001 vs. control group; ^#^ *p* < 0.05, ^##^ *p* < 0.01, ^###^ *p* < 0.001 vs. TNF-α group. Results are expressed as mean ± SEM (*n* = 3).

The cell viability was measured after incubation with various concentrations of HT-AC (12.5, 25, 50, 100 and 200 μmol/L) for 13 h using the CCK8 assay. Consistent with our previous report, we found that there was no significant difference in the cell viability of HT-AC groups for different concentrations as compared with the control group (Figure 3A). To further investigate the effect of HT-AC on pyroptosis, the HUVECs were pretreated with different concentrations of HT-AC (25, 50 and 100 μmol/L) for 1 h and then stimulated with TNF-α (40 ng/ml) for 12 h. We found that TNF-α increased the protein expressions of activated caspase-1 and caspase-1 activity. However, HT-AC significantly decreased the protein expressions of activated caspase-1 and caspase-1 activity compared with the TNF-α group (Figures 3B-D). HT-AC also significantly decreased the protein expressions of GSDMD (Figure 3E) and GSDMD-N (Figure 3F) compared with the TNF-α group. Moreover, HT-AC significantly suppressed TNF-α-induced the release of pro-inflammatory IL-1β (Figure 3G) and IL-6 (Figure 3H). We further found that HT-AC significantly decreased TNF-α-induced pore formation and membrane rupture by decreasing the release of LDH (Figure 3I) and the number of PI positive cells (Figure 3J).

### HT-AC Down-Regulated HDAC11 Expression in the Aortic Intima of HFD-Fed ApoE^−/−^ Mice and TNF-α-Stimulated HUVECs

**FIGURE 4:**
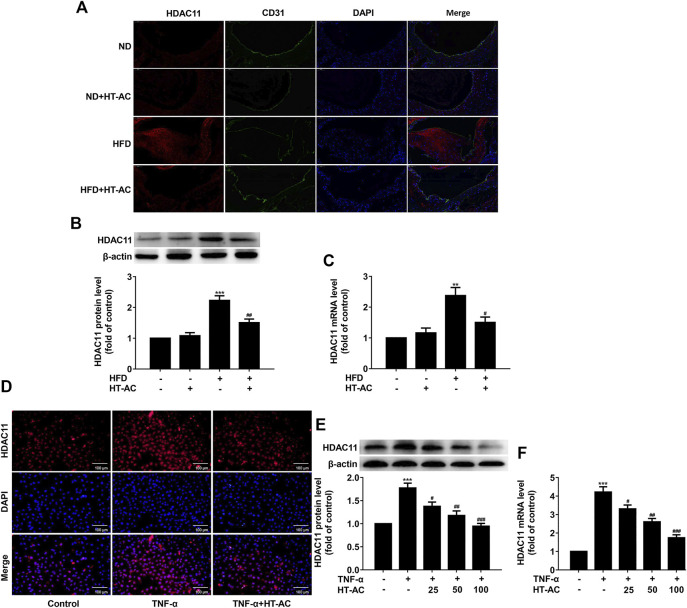
HT-AC down-regulated HDAC11 expression **(A)** The expression of HDAC11 in the aortic intima of ApoE^−/−^ mice by immunofluorescent double staining of the aortic sinus of HDAC11 and CD31. Scale bar indicates 50 μm **(B,C)** The expression of HDAC11 in the aorta of ApoE^−/−^ mice was determined by Western blotting and quantitative real-time PCR. **p < 0.01, ***p < 0.001 vs. ND group; ^#^ *p* < 0.05, ^##^ *p* < 0.01 vs. HFD group. Results are expressed as mean ± SEM (n = 10) **(D)** The expression and localization of HDAC11 in TNF-α-stimulated HUVECs were determined by immunofluorescence, ×200. Scale bar indicates 100 μm **(E,F)** The expression of HDAC11 in TNF-α-stimulated HUVECs was determined by Western blotting and quantitative real-time PCR. ***p < 0.001 vs. control group; ^#^ *p* < 0.05, ^##^ *p* < 0.01, ^###^ *p* < 0.001 vs. TNF-α group. Results are expressed as mean ± SEM (*n* = 3).

To investigate the effect of HT-AC on HDAC11 expression, we examined the expression of HDAC11 in the aortic intima of ApoE^−/−^ mice by immunofluorescent double staining of the aortic sinus of HDAC11 and CD31. The results showed that HDAC11 protein expression in the aortic intima of HFD-fed ApoE^−/−^ mice were increased as compared with the ND group, while treatment with HT-AC decreased HDAC11 protein expression (Figure 4A). We further examined the expression of HDAC11 in the aorta of ApoE^−/−^ mice by Western blotting and quantitative real-time PCR. We found that HDAC11 protein and mRNA expressions in the aorta of HFD-fed ApoE^−/−^ mice were significantly increased as compared with the ND group, while HT-AC significantly decreased HDAC11 protein and mRNA expressions compared with the HFD group (Figures 4B,C).

To further confirm whether the HDAC11 signaling pathway was involved in the regulatory effect of HT-AC on pyroptosis, HUVECs were pretreated with HT-AC for 1 h and then stimulated with TNF-α (40 ng/ml) for 12 h. The expression of HDAC11 was determined by immunofluorescence, Western blotting and quantitative real-time PCR. The results showed that HT-AC decreased HDAC11 protein and mRNA expressions compared with the TNF-α group (Figures 4D-F).

### HT-AC May Target HDAC11

**FIGURE 5:**
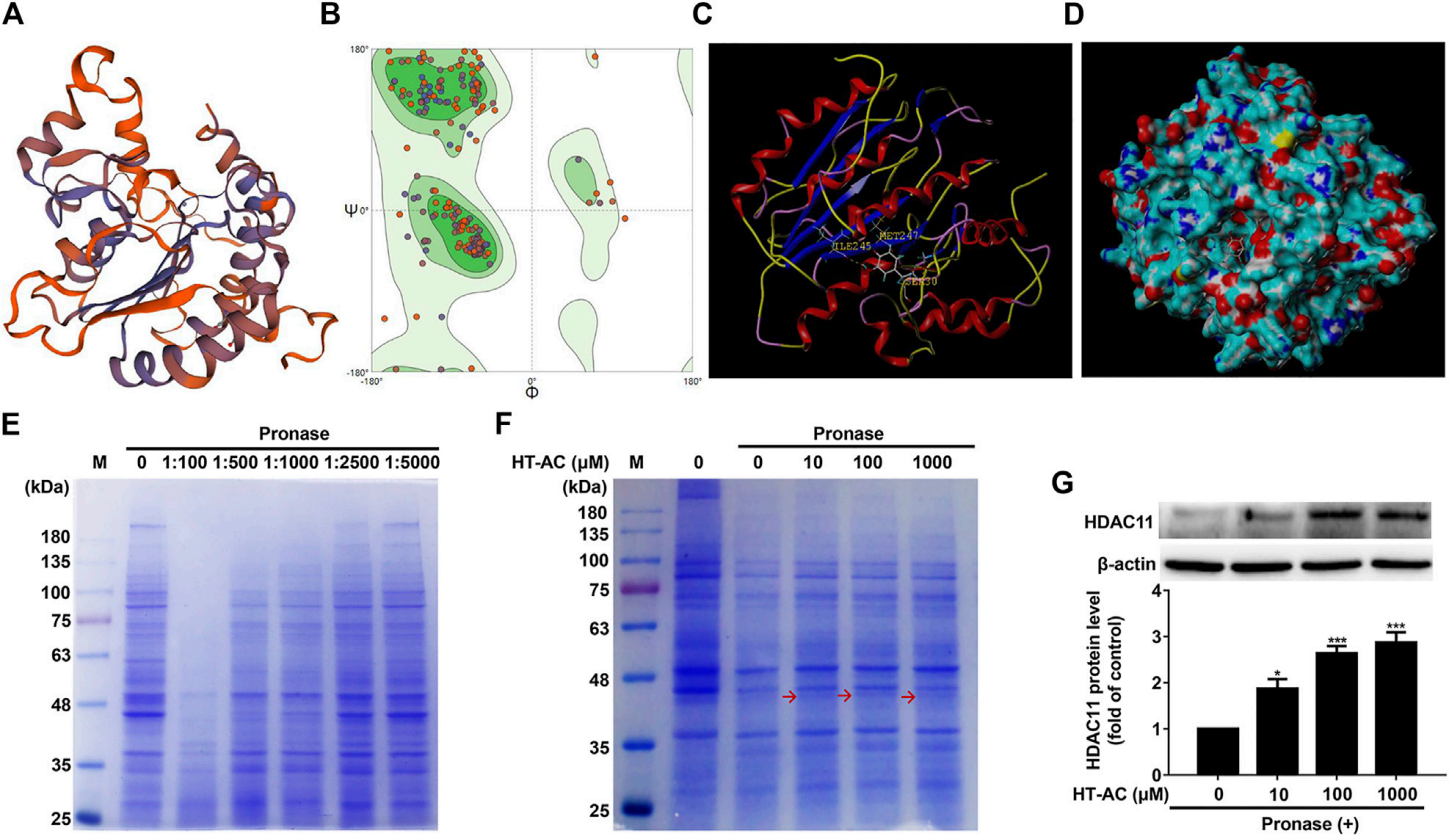
HT-AC may target HDAC11 **(A)** Schematic SWISS-MODEL model of human HDAC11 **(B)** Ramachandran plot analysis of the three-dimensional structure of human HDAC11. The green, light green, and light blue regions represent the “most favored”, “additional allowed” and “generously allowed” regions that amino acid residue fall regions, respectively **(C,D)** The ability of HT-AC to bind to the HDAC11 ligand-binding domain by molecular docking **(E)** Cell lysates were subjected to pronase digestion at various mass ratios of pronase to protein (1:100, 1:500, 1:1,000, 1:2,500 and 1:5,000) and coomassie staining **(F)** After treatment with HT-AC (10, 100 and 1,000 μmol/L) for 2 h at 4°C, cell lysates were subjected to pronase digestion at the 1:1,000 pronase to protein mass ratio and coomassie staining **(G)** The protein expression of HDAC11 was examined by Western blotting in HUVECs following HT-AC treatment. *p < 0.05 and ***p < 0.001 vs. control group. Results are expressed as mean ± SEM (*n* = 3).

Given our findings that HT-AC down-regulated HDAC11 expression in the aortic intima of HFD-fed ApoE^−/−^ mice and TNF-stimulated HUVECs, we explored the possibility of whether HT-AC targets HDAC11. Here, we evaluated the ability of HT-AC to bind to the HDAC11 ligand-binding domain to activate HDAC11 by molecular docking. The three-dimensional structure of human HDAC11 was built by homology-modeling (Figure 5A). Ramachandran plot analysis showed that the amino acid residue of the HDAC11 model had 91.27% in the most favored regions (Figure 5B). The molecular docking result showed good compatibility between HT-AC and HDAC11, indicating that HT-AC might act through HDAC11 (Figures 5C,D). DARTS methods were used to identify the potential molecular targets of HT-AC. We added pronase solutions at different mass ratios of pronase to protein (1:100, 1:500, 1:1,000, 1:2,500 and 1:5,000) to the experiment, the results showed that the 1:1,000 pronase to protein mass ratio was most suitable (Figure 5E). Further analysis revealed one visible differential band between HT-AC-treated HUVECs and the control group about 35–48 kDa molecular weight (Figure 5F). We also examined the protein expression of HDAC11 in HUVECs after treatment with HT-AC by Western blotting. Our results showed that various concentrations of HT-AC (10, 100 and 1,000 μmol/L) treatment of HUVECs prevented enzymatic digestion of HDAC11 protein compared with vehicle-treated pronase (Figure 5G).

### HT-AC Inhibited Pyroptosis Through HDAC11 in TNF-α-Induced HUVECs

**FIGURE 6:**
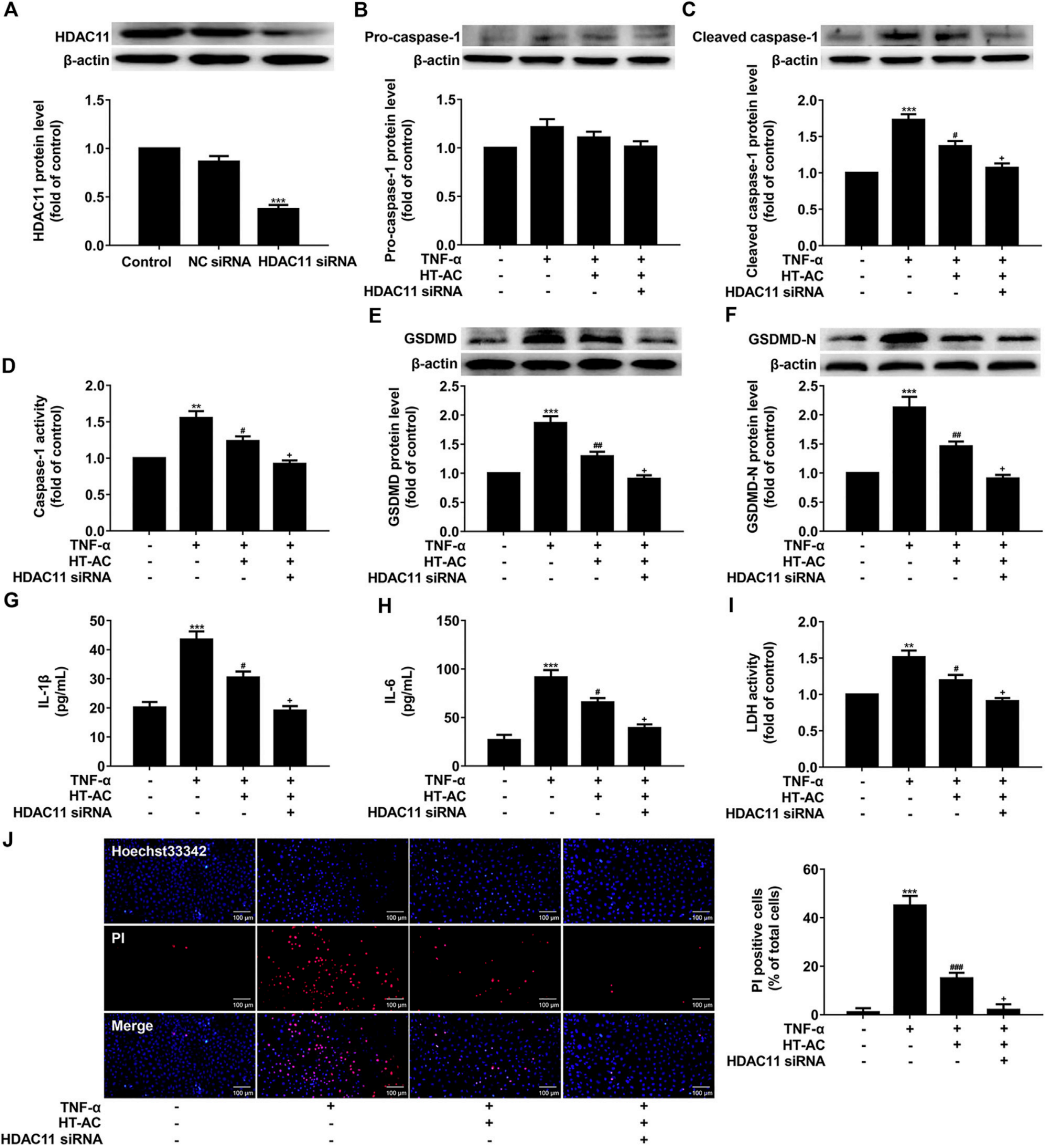
HT-AC inhibited pyroptosis through HDAC11 in TNF-α-induced HUVECs. HUVECs were transfected with HDAC11 siRNA or NC siRNA for 48 h **(A)** The expression of HDAC11 was determined by Western blotting. The HUVECs transfected with HDAC11 siRNA or NC siRNA were pretreated with HT-AC before stimulation with TNF-α **(B,C)** The protein expressions of pro-caspase-1 and cleaved caspase-1 were determined by Western blotting **(D)** Caspase-1 activity was assayed using the caspase-1 activity assay kit **(E,F)** The protein expressions of GSDMD and GSDMD-N were determined by Western blotting **(G,H)** The concentrations of IL-1β and IL-6 in cellular supernatant of HUVECs were detected by ELISA assay **(I)** The LDH release was evaluated with a cytotoxicity detection LDH kit **(J)** Double-fluorescent staining with PI (red) and Hoechst 33,342 (blue) was used to assess the formation of membrane pores during pyroptosis, ×200. Scale bar indicates 100 μm **p < 0.01, ***p < 0.001 vs. control group; ^#^ *p* < 0.05, ^##^ *p* < 0.01, ^###^ *p* < 0.001 vs. TNF-α group; ^+^ *p* < 0.05 vs. TNF-α+HT-AC group. Results are expressed as mean ± SEM (*n* = 3).

Given our findings that HT-AC down-regulated HDAC11 expression and HT-AC might target HDAC11, we further explored the possibility of whether the inhibitory effect of HT-AC on pyroptosis is related to HDAC11. HUVECs were transfected with HDAC11 siRNA or NC siRNA for 48 h, and then the protein expression of HDAC11 was examined by Western blotting. HDAC11 protein expression was significantly declined and the knockdown efficiency of HDAC11 was 63% (Figure 6A). Then the HUVECs transfected with HDAC11 siRNA were pretreated with HT-AC (50 μmol/L) before stimulation with TNF-α (40 ng/ml). The protein expressions of pro-caspase-1, cleaved caspase-1 were detected by Western blotting, and caspase-1 activity was assayed using the caspase-1 activity assay kit. The results showed that knockdown of HDAC11 increased the inhibitory effects of HT-AC on cleaved caspase-1 protein expression and caspase-1 activity in TNF-stimulated HUVECs (Figures 6B-D). In addition, knockdown of HDAC11 increased the inhibitory effects of HT-AC on the protein expressions of GSDMD and GSDMD-N in TNF-stimulated HUVECs (Figures 6E,F). Our results further showed that knockdown of HDAC11 increased the inhibitory effects of HT-AC on the cellular supernatant concentrations of IL-1β (Figure 6G), IL-6 (Figure 6H) and LDH (Figure 6I), and the number of PI positive staining cells (Figure 6J) in TNF-stimulated HUVECs. Taken together, our findings suggested that the modulatory effect of HT-AC on pyroptosis is partly mediated by HDAC11 in TNF-α-stimulated HUVECs.

## Discussion

HT-AC is present in the olive oil of the fruit and leaf of the olive. In the past few years, the beneficial effects of HT-AC have been demonstrated such as antioxidant (Gordon et al., 2001), antiplatelet aggregating (González-Correa et al., 2008), and anti-inflammatory effects (Rosillo et al., 2015) in CVDs. Herein, we demonstrated that HT-AC attenuated the formation of atherosclerotic lesions and inhibit pyroptosis in the aortic intima of HFD-fed ApoE^−/−^ mice, and HT-AC inhibited pyroptosis in TNF-α-stimulated HUVECs. We demonstrated that HT-AC might play a role in inflammation by regulating pyroptosis in endothelial cells to prevent and treat AS. Importantly, our results showed that HT-AC might prevent pyroptosis in vascular endothelial cells via down-regulation of HDAC11 related signaling pathway.

The studies have demonstrated that HT-AC exerted anti-inflammatory effects in CVDs (Tabernero et al., 2014; Rosillo et al., 2015). In the present study, we observed that HT-AC attenuated the formation of atherosclerotic lesions in HFD-fed ApoE^−/−^ mice. Previous studies have shown that treatment with salidroside (Xing et al., 2020) or Melatonin (Zhang et al., 2018) decreases atherosclerosis plaque formation in the aorta of HFD-fed ApoE^−/−^ mice via inhibiting endothelial cell pyroptosis. Pyroptosis is accompanied by the release of a large number of pro-inflammatory factors (Chang et al., 2013). It has been reported that dietary HT-AC inhibited TNF-α and IL-1β levels in the plasma of cholesterol-rich diet-induced hypercholesterolemic rats (Tabernero et al., 2014). Our previous study found that HT-AC inhibited TNF-α and IL-1β levels in the serum of hypercholesterolemic mice (Yao et al., 2019). In the present study, we observed that HT-AC inhibited pro-inflammatory TNF-α and IL-1β levels in the serum of HFD-fed ApoE^−/−^ mice. Several studies have provided evidence that pyroptosis is involved in the inflammatory process of AS (Chang et al., 2013). GSDMD N-terminal segment, the central effector molecule and executor protein, executes pyroptosis by promoting the formation of membrane pores (Liu H. et al., 2020). In the present study, GSDMD N-terminal segment was remarkably inhibited by HT-AC in the aorta of HFD-fed ApoE^−/−^ mice. GSDMD N-terminal segment is the cleavage product of activated caspase-1 (Liu Z. et al., 2020). We found that HT-AC treatment significantly suppressed the protein expression of activated caspase-1 in the aortic intima of HFD-fed ApoE^−/−^ mice. Combined with the above results, beneficial effects of HT-AC in the prevention and reversal of AS may be dependent on the regulation of activated caspase-1 and GSDMD signaling pathway. It has been well-known that TNF-α induces endothelial cell inflammatory injury, apoptosis and endothelial dysfunction (Jain et al., 2007). It has been indicated that TNF-α induced caspase-1 expression in A549 cells (Jain et al., 2007), and induced activation of caspase-1 in 3T3-L1 cells (Furuoka et al., 2016). During pyroptosis, GSDMD cleavage was dependent on caspase-1 activation, which promoted the formation of membrane pores and cell lysis as well as the release of pro-inflammatory intracellular contents (Opdenbosch and Lamkanfi, 2019; Liu Z. et al., 2020). In the present study, we observed that HT-AC treatment of HUVECs alleviated TNF-α-induced pyroptosis by decreasing the number of PI positive cells, decreasing the enhanced protein expression of activated caspase-1 and gasdermin D, as well as by decreasing the release of pro-inflammatory IL-1β and IL-6. These results suggested that HT-AC may play an anti-atherosclerosis role by inhibiting the pyroptosis of endothelial cells.

Histone deacetylase enzymes regulate diverse biological function, including gene expression, rendering them potential targets for intervention in a number of diseases (Moreno-Yruela et al., 2018). Among the human zinc-dependent histone deacetylase enzymes, the most recently discovered member, HDAC11, has been shown to play a role in CVDs through promoting apoptosis, oxidative stress and inflammation (Zhou et al., 2017; Fan et al., 2018). A study showed that peritoneal injection of a small-molecule HDAC11 inhibitor significantly attenuated the formation of neointima after carotid artery ligation in Sprague–Dawley rats (Fan et al., 2018). Another study showed that HDAC inhibitor Trichostatin A mitigated the inflammation-induced pyroptosis and apoptosis during endotoxemia-induced acute lung injury (Samanta et al., 2018). Our present study revealed that treatment with HT-AC decreased HDAC11 protein expression in aortic intima, and suppressed HDAC11 protein and mRNA expressions in the aorta of HFD-fed ApoE^−/−^ mice. In addition, it has been reported that TNF-α can upregulate the expression of HDAC11 in B cells in a dose-dependent manner (Shao et al., 2018). Our study also indicated that TNF-α can upregulate the protein and mRNA expressions of HDAC11 in HUVECs, while HT-AC decreased HDAC11 protein and mRNA expressions in TNF-α-stimulated HUVECs. Therefore, we further investigated the possibility of whether HT-AC targets HDAC11. The molecular docking result showed good compatibility between HT-AC and HDAC11. DARTS has been used for the identification of ligand targets by identifying the alterations in proteolytic sensitivity that occur after the binding of ligand and receptor (Gong et al., 2013; Pai et al., 2015). In our present study, the DARTS result showed that various concentrations of HT-AC (10, 100 and 1,000 μmol/L) treatment of HUVECs prevented enzymatic digestion of HDAC11 protein compared with vehicle-treated pronase. However, the differential band about 35–48 kDa molecular weight is very weak and there is no evidence that this band originated from HDAC11. To further elucidate this question, liquid chromatography-mass spectrometry (LC-MS) analysis will be conducted to confirm the binding between HT-AC and HDAC11 in our laboratory. In addition, whether the activities of HDAC11 are inhibited by HT-AC were not further analyzed in the present study. Moreover, these data do not exclude the possibility of other potential targets (Yang et al., 2017). Therefore, the molecular docking and DARTS analysis indicated that HT-AC may bind to HDAC11. In addition, Stammler D *et al.* revealed that treatment of HDACi increased lipopolysaccharide-mediated secretion of IL-1β via a caspase-1-independent mechanism (Stammler et al., 2015). Our research found that knockdown of HDAC11 with siRNA increased the inhibitory effects of HT-AC on pyroptosis in TNF-α-stimulated HUVECs. Therefore, HT-AC may exert the modulatory effect on pyroptosis partly through the HDAC11-mediated signaling pathway.

**FIGURE 7:**
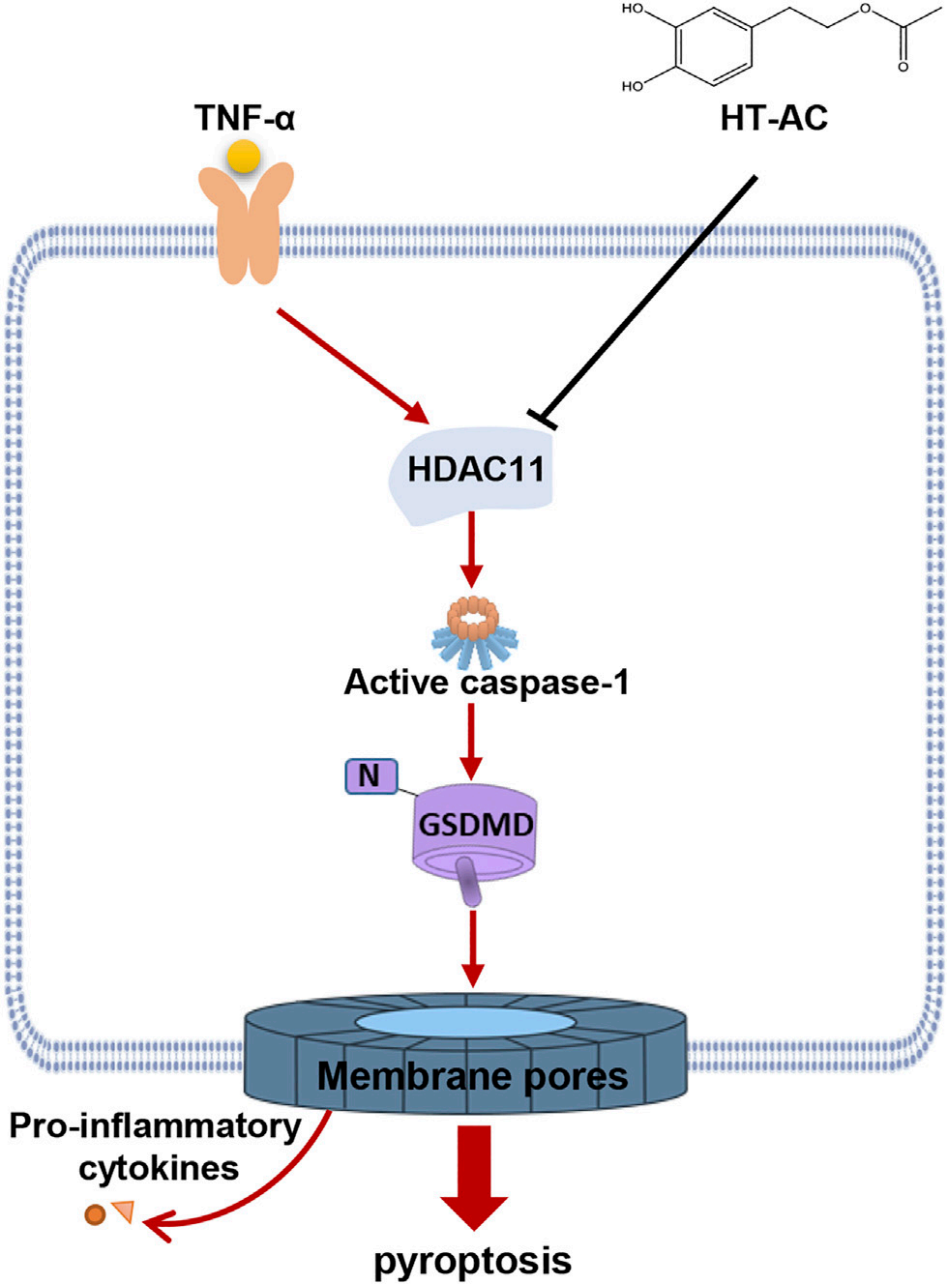
Schematic illustration of the signaling pathway involved in the effect of HT-AC on pyroptosis in HUVECs.

In conclusion, HT-AC *in vivo* attenuated the formation of atherosclerotic lesions and pyroptosis in the aortic intima of HFD-fed ApoE^−/−^ mice. *In vitro*, we found that HT-AC treatment of HUVECs alleviated TNF-α-induced pyroptosis. Moreover, HT-AC might prevent pyroptosis in vascular endothelial cells via down-regulation of HDAC11 related signaling pathway (summarized in Figure 7). These findings provide a novel mechanism to the beneficial effects of HT-AC in the prevention and reversal of AS.

## Data Availability

The raw data supporting the conclusions of this article will be made available by the authors, without undue reservation.
